# Physiological Proving of Lachesis Trig. Six Million (Fincke)

**Published:** 1885-03

**Authors:** B. Fincke

**Affiliations:** Brooklyn, N. Y.


### PHYSIOLOGICAL PROVING OF LACHESIS TRIG. SIX MILLION (FINCKE).

By DR. BUCHMANN, in *Alvensleben*, translated from the *Leipzig Allg. Hom. Zeitung*, Vol. 109, Nos. 18–22.

B. FINCKE, BROOKLYN, N. Y.

Mrs. F. B., æt. fifty four. May 16th, 1884, takes the vial with globules of Lachesis 6M (Fincke) in her right hand at 7.40 A. M. After four minutes, collection of water in mouth and nose; frequent yawning, with lacrymation; grumbling motion in the intestines in the umbilical region; urging to stool; continued drawing pain from both hypochondria toward the navel; frequent eructations; wound-like sensation in the anterior part of the nostrils; sensation of swelling in the pharynx, rendering the swallowing of saliva difficult; pain in the masticatory muscles, caused apparently by violent yawning. Twenty-five minutes after holding the vial, the yawning becomes so spasmodic that she cannot bear it any more and must lay the vial aside. The above symptoms have been observed alternating till then.

At 8.05 A. M., the symptoms disappeared gradually after the removal of the vial, only the eructations last till 11.45 A. M. Then pressure in the pit of the stomach as of a great stone, not preventing her from eating; wound-like sensation in the cavity of the nose anteriorly; great lassitude for one hour after 8.05 A. M. Since February 27th a regular evacuation every morning and evening had taken place. After the evacuation this morning at the usual time, a solid evacuation followed; a second a few hours after the first, at 9 A. M., and a third one at 10.30 A. M., the last one moderate, with some burning in anus.

3.30 P. M.—Since noon, frequent pressure in the pit of the stomach and on the lower end of the breastbone, as of a heavy load. At the same time, on the same level, a pain in the back when taking a full breath, as large in circumference as a hand, a symptom never before noticed. At supper, increased appetite. The usual evacuation in the evening did not appear.

May 17th.—Morning. She has not slept at all in the night till 3.30 A. M., though she went to bed very tired. Toward 11 P. M., restlessness drove her out of bed. She could not keep her hands and feet still, and cannot stay in one place (similar symptoms as she once had when proving Chelidonium). This was repeated three times during the night. She had to walk about for fifteen to thirty minutes each time, with groaning, fear, and anxiety (otherwise she enjoys very good sleep). During the night several times attack of dry cough and drawing pain from both hypochondria toward the umbilicus. No evacuation this morning.

9 A. M.—Great lassitude since rising, then rush of blood to the head, with confusion. Margins of nostrils red, sore, and painful.

10.45 A. M.—She must lie down, being so weak; sensation of swelling in the vertex, with violent boring and digging pain in the brain with groaning for one hour, removed by laying on of hand and mesmeric passes from the vertex down to the ends of the fingers, as the fitting antidote of Lachesis.

7 P. M.—From 2 to 7 P. M., spasmodic drawing from both hypochondria toward the umbilicus, where it caused a sensation as if an evacuation would follow, alternating with stitches in the margin of the right lower ribs in the axillary line, and pain in the back as described above, forcing her to lie down several times; great lassitude and tiredness till going to bed at 10 P. M. No evacuation has taken place yet.

May 18th.—After midnight, good sleep till 5 A. M. On rising, weakness, with stitches in the right side from 5 to 6 P. M. Again no evacuation this morning.

12 M.—Till noon, rumbling about the umbilicus with urging to stool every half hour or forty-five minutes. Appetite good. No evacuation in the evening. Abdomen hard and much distended by accumulation of wind in the colon.

May 19th.—No evacuation neither in the morning nor in the evening. Abdomen hard, distended by flatus.

May 20th.—In the morning, scanty, hard stool, with painful sensation in the head and sensation of distention in the vertex, as if by enlargement of the brain the skull were driven asunder in an upward direction. Sensation as if the anus were firmly closed. Nostrils still sore, from May 17th till to-day.

May 21st.—Evacuation morning and evening.

May 22d.—Very copious pappy stool in the morning and evening.

All through the month of June, feeling well as before the proving.

### EPICRISIS.

Even if we should expect ever so slight a practical use from the highest fluxion potency which so far has been made, yet no unbiased observer will lay aside the inductive provings of Lachesis with high potencies without satisfaction when he has given attention to my scientific investigations for the explanation of the law of Simility. I, of course, reject the acknowledgment of those who subordinate their judgment unconditionally to the dicta of the scholastic guild in a territory which is entirely foreign to it. The latest history of Homœopathy, however, has furnished the proof that the descent to low attenuations, owing to the doubt in the efficacy of high potencies, has only damaged the respectability of Homœopathy. The sharper we place the contrasts, the more compact we will stand against our opponents, but with giving up our infinitesimal doses we would give up Homœopathy itself. I attach some value to these provings of Lachesis, because they are well suited to strengthen the confidence in high potencies and to stimulate experimentation. But where, as in the foregoing investigations, observations going against old-fashioned notions and apparently inexplicable facts are concerned, one wants to see with one's own eyes or to be able to trust to the observations of others unconditionally before forming a judgment about it. Our whole Materia Medica Pura is founded upon the trust we put in the truthfulness and conscientiousness of our provers, and I can warrant these qualities in my trusted prover.

Observations of cures with the highest potencies are not scarce, and I myself have had the good fortune to obtain one with striking rapidity by Phosphorus CM (Fincke). See HOMŒOPATHIC PHYSICIAN, August, 1884, p. 222. Though this cure strikes one by its object being a chronic disease, in which there was no reliance to be had upon the *vis medicatrix naturæ,* yet the exact experimental method requires that we be not content with the general *post hoc ergo propter hoc* if the question to be decided is what a remedy has done. We, as homœopathicians, in contraposition to the allopathicians, are in the happy position to be able to base our radical healing method by removal of the morbific cause upon a natural law, in which the *similia similibus* forms a test for the rectitude of that decision, and that in such diseases which, experimentally, are not removed by the *vis medicatrix* of nature, and where the influence of other healing agents is excluded.

The two lastly observed symptoms, *soreness of the nostrils* and *closure of the anus, alone would have already sufficed to recognize the proved remedy as Lachesis if it had been unknown, because both symptoms are also found in the provings of Lachesis by Hering, and no other remedy has these two symptoms.* If now, in a disease (aside from the other symptoms) which shows these two symptoms, we obtain a cure after Lachesis, we have the sure proof of a healing with Lachesis, also with the six millionth potency, provided that a spontaneous recovery is excluded.

When, after an induction of twenty-five minutes' duration, the vial was removed and the yawning spasm immediately ceased, I thought that, as at the first proving, no further symptom would occur. I was also perfectly satisfied with my success so far, since this time even the symptoms of increased peristaltic action had appeared, and I had not expected that, probably by the longer time of induction, such a violent after-action, lasting for five days, would take place which the next day caused much anxiety on account of the brain-symptoms.

When comparing the first inductive proving of Lachesis 5M (Fincke)—see HOMŒOPATHIC PHYSICIAN, September, 1883, p. 260—with the preceding proving, the difference in the symptoms is at once apparent; then, after three minutes, stormy symptoms of short duration occurred, and a repetition of the proving the following day had no effect. The affinity for Lachesis was satisfied, or, as the senior of the conservative homœopathicians in the United States, Dr. P. P. Wells, had expressed it, the susceptibility for Lachesis was exhausted, and with it the affinity for morbific causes with *similar* weaker affinity (isopathic and preservative action; see HOMŒOPATHIC PHYSICIAN, April, 1883, p. 115).

The affinity for Lachesis 6M was, after a year, not yet extant in the respiratory organs in that degree as before the induction of Lachesis 5M, where already, after a few minutes of induction, violent yawning spasms, with sensation of suffocating, occurred. We must not attribute this difference in the action to a weaker effect of the higher potency, since at the last proving it also extended to the abdominal organs, and became more energetic and enduring, probably on account of the longer time of induction.

We always have been in the habit of using infinitesimal doses, but we have never thought that it would be possible to obtain pathopoëtic symptoms, and even objective ones, with the six millionth potency of a remedy, and that, by induction from globules contained in a closed vial, a method which, however, succeeds only with sensitives who are especially predisposed, since non-sensitives can carry a vial with such a high potency in their hands all day long without being in the least affected.

In order to bring this effect of the present high potency nearer to our contemplation, we for once must ignore the notion of matter altogether and institute a comparison with the physical forces, which are potentiated by addition of other physical forces, even when thus far they had been latent; the magnetism inherent in the loadstone can, by passes in a certain direction, be permanently transmitted to steel bars, and successively from these again upon others *ad infinitum*, without losing the capability of attracting iron filings even through other solid bodies. Everybody accepts this property of magnetism as self-understood, though it is perfectly incomprehensible. Thus also it is with the increase of the affinity of a magnet for iron by the known methods. Now, it would be an analogous process in the potentiation of medicines if we imagine that through the attenuation, by annihilating the *cohesive* force, this force in a measure is changed into *medicinal* force, that the medicinal substance lastly is transmitted into mere medicinal force, a process in which, by addition of still other *physical* forces (heat, friction, succussion, electricity), the medicinal force, possibly, is reproduced in a similar manner as the pathogenetic force of certain morbific substances is reproduced according to experience by the *vegetative* forces of certain micro-organisms in their multiplication.*

*Thus far it was thought that after discovery of the micro organisms for each infectious disease, also the cause of these diseases were discov-

*Dr. Koch*, in Philadelphia, has already (*Allg. Hom. Zeit.*, Vol. 96, No. 25,) developed his view, that motion, heat, electricity, and magnetism are disengaged as correlative forces in the potentiating process, and actually must show themselves while they saturate the atoms of the remedy with their own impenetrable forces. But this hypothesis would not explain the potentiation of the specific medicinal force, because it is wanting the basis of fact.

All observers who are not biased by prejudice, but, by studying extensive provings, have arrived at the point of being able to have a competent judgment, will agree with me that the symptoms detailed above can only be recognized as symptoms of Lachesis.

The prover had been quite well, and, since the cure by Phosphor. CM from the beginning of December, 1883, till May 16th, 1884, she had a regular evacuation every morning and evening. The same took place likewise May 16th, before 7 A. M. At 7.40 A. M. she took the vial of Lachesis 6M in her hand for twenty-five minutes. At 9 A. M. a second hard and at 10.30 A. M. a third watery evacuation, and then a constipation of four days' duration followed. *Nothing* besides has had any

---

ered in them, and means were sought for to kill those organisms, or at least to stem their development. I, in the beginning, have from the homœopathic standpoint, held on to the view that they are only the carriers of a special poison with specific affinity, such as the Acarus scabiei or the itch-poison. Now lately the discovery has been made in the Pathological Institute of Professor Semmer, in Dorpat, that the bacilli and micrococci of anthrax are the product of a special anthrax-virus. (*Allg. Med. Central Zeitung*, 1884, 47.) Rosenberger already had shown that by inoculation with boiled septic blood, free of micro-organisms, the symptom-complex of septicæmia was produced, and that in the animals experimented on, after perishing, the same micro-organisms had been found as in animals which had perished after injection of unboiled septic blood.

Animals inoculated with boiled anthrax-virus perished in three to six days, had the anthrax-bacilli in a quarter of the cases, in the rest the characteristic micrococci, and in all the first grades of development of the bacilli. The animals for control likewise inoculated and provided with protective inoculation, showed the next days no abnormity, except an increase of temperature by 1.5 C. Osol says about this: "This fact, by itself alone, even if no typical micro-organisms were found in the blood of the animals which perished in consequence of the injection of boiled anthrax-blood, would clearly indicate a chemical anthrax-poison. The bacilli do not represent the primary but the secondary condition, and receive their virulence only after the influence of an inorganic chemical poisonous substance."

*My scientific explanation of the law of simility, which is incompatible with the assumption of a primary contagium vivum, finds an unexpected confirmation by these discoveries, since here it is proved by experiment that by the stronger affinity for the attenuated latent simillimum induced in the same way, the affinity for the same poison, fatal by too massy an incorporation, is neutralized, and in this manner a safe preservative is gained against it.*

influence, also no mental emotion, by which these irregularities could have been occasioned. After cessation of the symptoms, May 20th, the evacuation became as regular as before, and so it has been ever since. This action upon the peristaltic motion suffices for itself to remove every possible doubt as to the connection between cause and effect in this proving.

I may be excused from giving the further explanation of the physiological connection of the symptoms above enumerated, since they fit entirely into the pathopoëtic picture of *Hering* in supplying and completing it. Our pathopoëtic picture contains forty-two symptoms from the thirtieth potency.

In conclusion, I beg to present the symptoms obtained by induction of the highest fluxion potencies according to the usual schema, in the conviction that high-potency symptoms will mostly prove decisive in the therapy of chronic diseases.

*Ceterum censeo macrodosiam esse delendam.*

